# Smartphone-Based Cardiac Rehabilitation Program: Feasibility Study

**DOI:** 10.1371/journal.pone.0161268

**Published:** 2016-08-23

**Authors:** Heewon Chung, Hoon Ko, Tharoeun Thap, Changwon Jeong, Se-Eung Noh, Kwon-Ha Yoon, Jinseok Lee

**Affiliations:** 1 Department of Biomedical Engineering, Wonkwang University College of Medicine, Iksan, 54538, Republic of Korea; 2 Imaging Science based Lung and Bone Disease Research Center, Wonkwang University, 460 Iksandeaero, Iksan, Jeonbuk, 54538, Republic of Korea; 3 Department of Rehabilitation Medicine, Wonkwang University Colledge of Medicine, Iksan, 54538, Republic of Korea; 4 Department of Radiology, Wonkwang University Colledge of Medicine, Iksan, 54538, Republic of Korea; Medizinische Universitat Innsbruck, AUSTRIA

## Abstract

We introduce a cardiac rehabilitation program (CRP) that utilizes only a smartphone, with no external devices. As an efficient guide for cardiac rehabilitation exercise, we developed an application to automatically indicate the exercise intensity by comparing the estimated heart rate (HR) with the target heart rate zone (THZ). The HR is estimated using video images of a fingertip taken by the smartphone’s built-in camera. The introduced CRP app includes pre-exercise, exercise with intensity guidance, and post-exercise. In the pre-exercise period, information such as THZ, exercise type, exercise stage order, and duration of each stage are set up. In the exercise with intensity guidance, the app estimates HR from the pulse obtained using the smartphone’s built-in camera and compares the estimated HR with the THZ. Based on this comparison, the app adjusts the exercise intensity to shift the patient’s HR to the THZ during exercise. In the post-exercise period, the app manages the ratio of the estimated HR to the THZ and provides a questionnaire on factors such as chest pain, shortness of breath, and leg pain during exercise, as objective and subjective evaluation indicators. As a key issue, HR estimation upon signal corruption due to motion artifacts is also considered. Through the smartphone-based CRP, we estimated the HR accuracy as mean absolute error and root mean squared error of 6.16 and 4.30bpm, respectively, with signal corruption due to motion artifacts being detected by combining the turning point ratio and kurtosis.

## Introduction

The incidence of heart disease shows a trend of a continuous increase due to aging of the population, high blood pressure, diabetes, smoking, psychological stress, changes in lifestyle, chronic fatigue, and lack of exercise. The World Health Organization (WHO) has reported that approximately 17 million people die due to cardiovascular disease every year [1,2]. Owing to the increase in mortality due to heart disease, there is an urgent need to reduce the number of such deaths and to help patients return to a normal life in a timely manner. The American Heart Association (AHA) has shown that active participation in a cardiac rehabilitation program (CRP) after cardiac disease is effective for lowering the recurrence rate of cardiac disease, indicating the importance of engaging in a CRP [1,2]. CRPs are now widely used worldwide and incorporated into the infrastructure of hospitals.

Despite its reported benefits, the rate of outpatient participation in CRPs has been shown to be low after hospital discharge because of time constraints regarding hospital visits and the economic burden of attending [3–6]. Recently, research on the effectiveness of home-based or community-based exercise programs has been performed by comparing them to hospital-based CRPs; is no difference in effectiveness between them was observed, especially regarding the rate of recurrence of cardiac disease. During a CRP, the intensity of exercise is important because the exercise has to be appropriate, i.e., not too strenuous and not too relaxed. Exercise intensity is determined based on the measured heart rate (HR). For a given target heart rate zone (THZ), if the measured HR is greater than the THZ, the exercise intensity is too high and should be reduced. On the other hand, if the measured HR is less than the THZ, the exercise is inefficient, and the patient needs to exercise more intensively. Thus, during a CRP, measuring HR is the most important factor to monitor the patient’s exercise intensity. However, in home-based exercise, HR-measuring equipment such as electrocardiography (ECG) is not available and efficient exercise cannot be performed based on the CRP guidelines [2,5,7,8]. Therefore, there is a need for an HR-measurement-based CRP that is readily accessible, relatively inexpensive, and simple to operate, to achieve wide acceptance without requiring help from medical staff [8,9]. The proliferation of smartphones is relevant in this context because they meet the criteria of easy access and acceptance [5,9–11]. In addition, smartphones have the capacity to estimate HR by using video images of a fingertip taken with the built-in camera.

In this paper, we introduce a smartphone-based cardiac rehabilitation program (S-CRP) that utilizes only a smartphone, with no need for any external devices. For efficient cardiac rehabilitation exercise, we developed an application to automatically guide the intensity of the exercise by comparing the estimated HR with the THZ. The HR was estimated by using video images of a fingertip taken with the smartphone’s built-in camera. The S-CRP was developed using the Objective-C programming language. The introduced S-CRP includes pre-exercise, exercise with intensity guidance, and post-exercise. In the pre-exercise stage, exercise information, such as the THZ, exercise type, exercise stage order, and duration of each stage, is set up. In the exercise with intensity guidance, the S-CRP estimates HR via determination by the smartphone, and compares the estimated HR with the THZ. Based on this comparison, the S-CRP adjusts the exercise intensity to shift the patient’s HR to the THZ during exercise. In the post-exercise stage, the S-CRP provides the ratio of the estimated HR to THZ and a questionnaire on factors such as chest pain, dyspnea, and leg pain during exercise, which can act as objective and subjective evaluation indicators.

## Method

### Ethics Statement

This study was approved by the institutional review board (IRB) of the Wonkwang University Hospital and all participants provided full written consent.

### Pre-Exercise on Cardiac Rehabilitation Program

For a successful CRP, it is important to determine the THZ, which can differ from patient to patient; the S-CRP requires input of the patient’s THZ. Clinically, THZ can be determined by the exercise tolerance test (ETT) [2]. Alternatively, it can be determined by the heart rate reserve (HRR), which is determined by a Karvonen formula considering age, resting HR (bpm), and target intensity (%) as follows [2]:

$$THZ=target\;intensity\times \left(HR_{max}-HR_{rest}\right)+HR_{rest}$$ (1)

Where *HR*_*max*_ is a maximum HR and *HR*_*rest*_ is a resting HR.

As shown in Table 1, the target intensity can be determined based on the intensity of the exercise that a patient intends to perform, or on the rating of perceived exertion (RPE), which is recommended by the American College of Sports Medicine (ACSM) guidelines [2,12,13]. HR_max_ can be calculated in two ways: 207 –(0.7 × age) for a healthy person who has regularly been performing exercise, and 220 –age for a person with a low physical fitness level or requiring cardiac rehabilitation [2,14,15]. There are also other ways to calculate HR_max_, with each different method having further applications [15].

**Table 1:** Intensity and HR_max_ of RPE [2,13].

Exercise Intensity	RPE	Target intensity (%)
Very, Very Light	6–8	< = 56
Very Light	9–10	57–60
Light	11–12	61–64
Moderate	13–14	70–76
Hard	15–16	81–86
Very Hard	17–18	91–96
Maximal	19–20	> = 97

To measure HR_rest_, the S-CRP asks the patient to place their finger on the camera lens for 2 s at rest. We recently successfully demonstrated that using a smartphone’s camera to image a fingertip pressed onto it yields pulsatile signals that are similar to HR fluctuations [16]. The Beer-Lambert law states that the absorption of light as it passes through a sample is proportional to the thickness and the concentration of the sample, as follows:

−dI∝I⋅C dx, (2)

Where *dI* is the infinitesimal change in light intensity as it passes through a sample of concentration *C* and thickness *dx*. Then, for a large sample,

$$\text{I}=I_{o}e^{-\alpha \cdot C\cdot x}$$ (3)

Where *I*_*o*_ is the intensity of the incident light and *α* is the absorption coefficient, and *x* is the thickness of the sample. The thickness of the finger artery fluctuates as the heart beats. Correspondingly, the intensity of reflected light also fluctuates with the HR. To determine the pulse, the subject places their finger on the smartphone’s camera so as to fully cover the camera lens. The light from the LED passes through the finger and the camera records the change in illumination. The volumetric change of blood in the finger changes the light absorption and thus can be used to produce a photoplethysmogram (PPG). Videos were recorded at a resolution of 1,280 × 720 pixels with a sampling rate of 30 frames per second (fps) for 2 s, where HR was calculated based on the 2-s pulse. In reality, the sampling rate changes slightly between 25 and 30fps due to the internal processing load. For the frame rate variability, we interpolated the pulsatile signal to 30Hz using a cubic spline algorithm followed by peak detection for HR estimation. The HR estimation algorithm incorporated a filter bank with variable cut-off frequencies, spectral estimates of the HR, rank-order nonlinear filters, and decision logic [17].

Once the THZ is set up, the patient chooses the exercise type, exercise stage order, and duration of each stage in the S-CRP. ACSM recommends exercise consisting of four stages: warm-up, main exercise, rest, and cool-down. The main exercise stage can be split into multiple short stages. If a patient wants only one main exercise stage, the exercise stage order can be warm-up, main exercise, and cool-down without rest. If a patient wants two main exercise stages, the exercise stage order can be warm-up, main exercise, rest, main exercise, and cool-down. For the warm-up and cool-down, walking or light stretching is recommended. The main exercise stage can be outdoor cycling, indoor cycling, use of a treadmill, jogging, strength training, stair climbing, and rowing [2]. Based on the above recommendation, we developed the S-CRP in a way that enables the patient to set up the exercise type, exercise stage order, and the duration of each stage. For the exercise type, the patient can select from among use of a treadmill, indoor cycling, and outdoor jogging. In addition, more specific information on the main exercise can be input (e.g. speed and slope for the treadmill, and speed for indoor cycling). Finally, the patient sets up the frequency of HR estimation (e.g., every 30 s), since it is difficult to continuously place a fingertip on the smartphone camera.

### Exercise in the Cardiac Rehabilitation Program

#### Automatic voice guidance

Given the pre-exercise information, the patient can exercise with a smartphone starting from warm-up to cool-down. During the exercise, voice guidance regarding exercise status, HR estimation, and exercise intensity is provided, as shown in Table 2. For the exercise status guidance, upon each stage transition, namely, warm-up to main exercise, main exercise to rest, rest to main exercise, and main exercise to cool-down, voice guidance is provided through the built-in microphone in the smartphone. For the HR estimation guidance, the message “Please place your fingertip on the camera to estimate your heart rate” is given to the patient to periodically estimate HR, which is based on a 2-s pulse signal from video images. Subsequently, the patient places their fingertip on the camera for 2 s to estimate the HR. However, the acquired signal can be corrupted by motion artifacts, resulting in inaccuracy in the estimated HR. In such cases, the estimated HR has to be discarded. Thus, in the S-CRP, the measured 2-s pulse signal is analyzed to check whether it has been corrupted by motion artifacts. The motion artifact detection algorithm is described in the following subsection. If a pulse signal is found to be corrupted by motion artifacts, the voice guidance provides the following message: “Your measured pulse is of low quality. Please place your fingertip on the camera again.” For the exercise intensity guidance, based on the comparison between the estimated HR and the THZ, voice guidance regarding the exercise intensity is provided to the patient. If the estimated HR is greater than the THZ, the voice guidance, “Your heart rate is high. Please slow down” is given to the patient. If the estimated HR is lower than the THZ, the voice says: “Your heart rate is low. Please speed up.” If the estimated HR is within the THZ, the voice says: “Your heart rate is within your target heart rate zone. Please maintain this pace.” In this way, the patient can maintain a level of exercise intensity that keeps the HR within the THZ.

**Table 2:** Voice guidance on exercise status, heart rate (HR) estimation and exercise intensity.

Guidance	Status	Voice guidance
**Exercise Status Guidance**	To start exercise (warm-up stage)	It is time to do warm-up exercise.
	To shift to the main exercise stage	It is time to do the main exercise.
	To shift to the rest stage	It is time to take a rest.
	To shift to the second main exercise stage	It is time to do the second main exercise.
	To shift to the cool-down stage	It is time to do cool-down exercise.
**HR Estimation Guidance**	To estimate HR	Please place your fingertip on the camera to estimate your heart rate.
	To detect motion artifact	Your measured pulse is of low quality. Please place your fingertip on the camera again.
**Automatic Exercise Intensity Guidance**	To slow down (measured HR > THZ)	Your heart rate is high. Please slow down.
	To maintain intensity (measured HR within THZ)	Your heart rate is within your target heart rate zone. Please maintain your pace.
	To speed up (measured HR < THZ)	Your heart rate is low. Please speed up.

THZ, target heart rate zone

#### HR estimation and motion artifact detection during exercise

During exercise, HR is periodically measured by the S-CRP, which asks the patient to place their finger on a camera lens for 2s. However, the pulsatile signal during exercise can be corrupted by motion artifacts because of change in the pressure or location of the fingertip on the camera lens. If the signal is corrupted by motion artifacts, HR cannot be accurately estimated. Thus, motion artifact detection is important for avoiding inaccurate HR estimation. In the S-CRP, if the measured 2-s pulse during exercise is found to be corrupted by motion artifacts, HR is no longer estimated and another 2-s pulse is measured.

To evaluate HR estimation and motion artifact detection during exercise with the S-CRP, we used a modified version of the Bruce protocol, which consists of 5 min of walking for a warm-up, 10 min of jogging, 5 min of rest, an additional 10 min of jogging, and 5 min of walking for cooling down, all on a treadmill. For the first session of jogging, the slope was 12° and the speed was 4.0 km/h. For the second session of jogging, the slope and speed were slightly increased to 13° and 5.4 km/h. For HR estimation, 32 subjects who presented for cardiac rehabilitation exercise at Wonkwang University Hospital were recruited by trained study personnel. Twenty men and twelve women with an average age of 34.4 ± 15.5 years were recruited. Our protocol for data collection and analysis on participants was approved by the Institutional Review Board of the Wonkwang University Hospital. The camera of an iPhone 6 (Apple Inc., Cupertino, CA, USA) was placed on either the left or the right index finger of the study participants for a total of 30 min.

**Fig 1:**
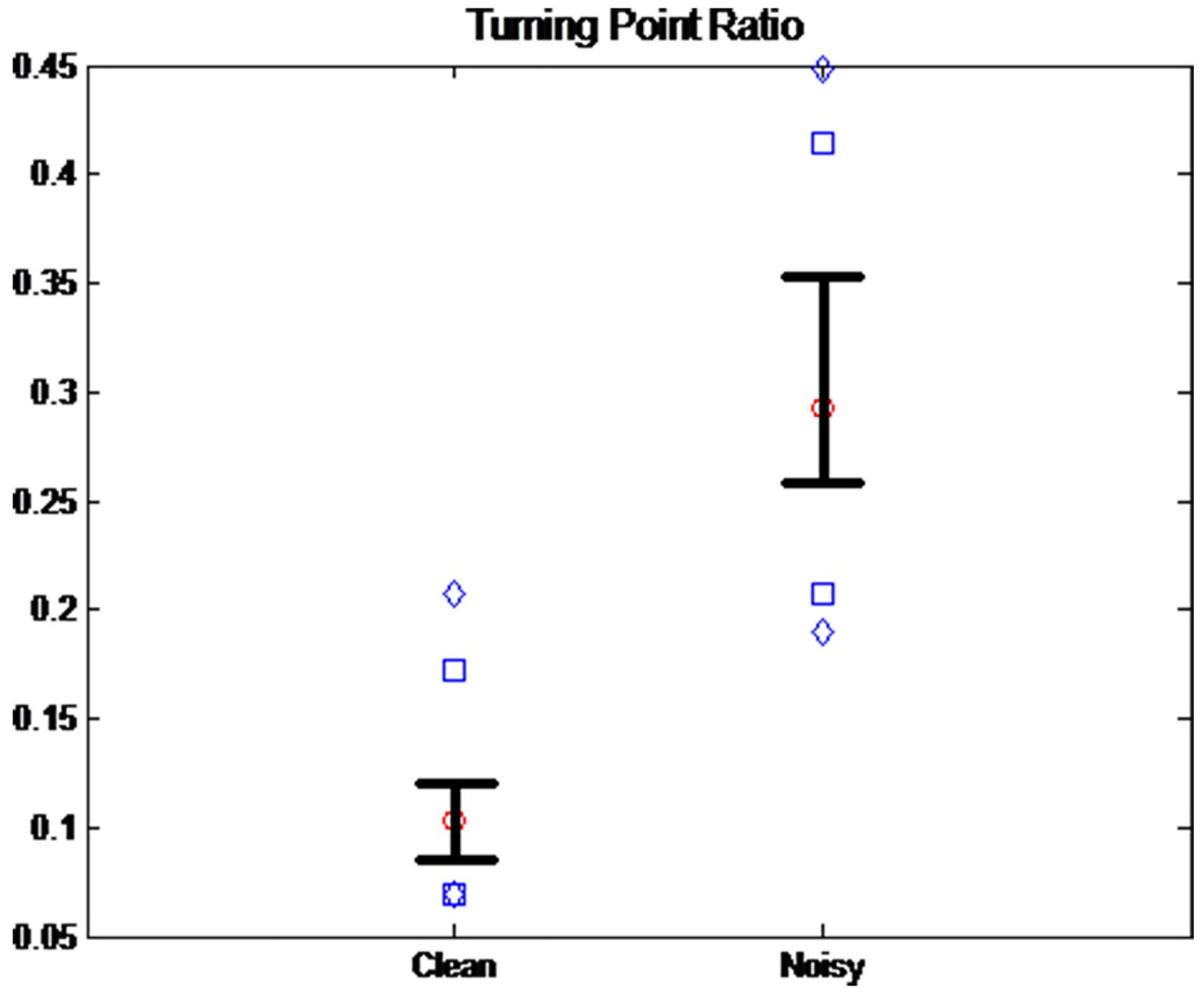
The distribution of the turning point ratio (TPR) in the clean and noisy signal groups. The diamonds above and below represent the 5^th^ and 95^th^ percentiles of each group, and the squares above and below represent the 90^th^ and 10^th^ percentiles. Whiskers above and below represent the 75^th^ and 25^th^ percentiles, respectively. The circle is the median value.

For motion artifact detection, we used two statistical methods: turning point ratio (TPR) and kurtosis. TPR is a useful tool to determine whether a temporal or spatial series is random [18]. Given three random numbers x_1_, x_2_ and x_3_, where x_1_ > x_2_ > x_3_, six possible series combinations can be expected. Among them, (x_1_ x_2_ x_3_), (x_2_ x_3_ x_1_), (x_2_ x_1_ x_3_) and (x_3_ x_1_ x_2_) include turning points, while (x_1_ x_2_ x_3_) and (x_3_ x_2_ x_1_) do not. For the TPR calculation, we counted the turning points for three successive samples and repeated until the end of the samples for the total number of turning points in the entire number of samples N. Finally, we divided the total number of turning points by N−2 for the TPR value. If the time series is random, TPR is expected to be higher than that of a sinusoidal wave. We investigated the values of TPR between the clean signals and noisy signals. The 2-s segments (N = 28,800) collected from the 32 recruited subjects during 30-min exercise were manually classified into clean and noisy signals by trained study personnel. One of the main criteria was whether the pulse peaks were recognizable. Fig 1 shows the distributions of TPR values from the clean and noisy signal groups. The diamonds above and below represent the 5^th^ and 95^th^ percentiles of each group, and the squares above and below represent the 90^th^ and 10^th^ percentiles. Whiskers above and below represent the 75^th^ and 25^th^ percentiles, respectively. The circle is the median value. The mean ± standard deviation was 0.1089 ± 0.0386 for a clean signal and 0.3047 ± 0.0837 for a noisy signal. This shows that the TPR mean value of the noisy signal is 2.80 times higher than that of the clean signal. In addition, there was a significant difference between the two groups at p < 0.01 based on ANOVA and Bonferroni’s t-test.

Kurtosis (Kur) is a measure of the data distribution, which is based on a scaled version of the fourth moment of the data, as follows:

$$Kur=\frac{\mu_{4}}{\sigma^{4}}=\frac{E\left[{\left(X-\mu \right)}^{4}\right]}{{\left(E\left[{\left(X-\mu \right)}^{2}\right]\right)}^{2}},$$ (4)

**Fig 2:**
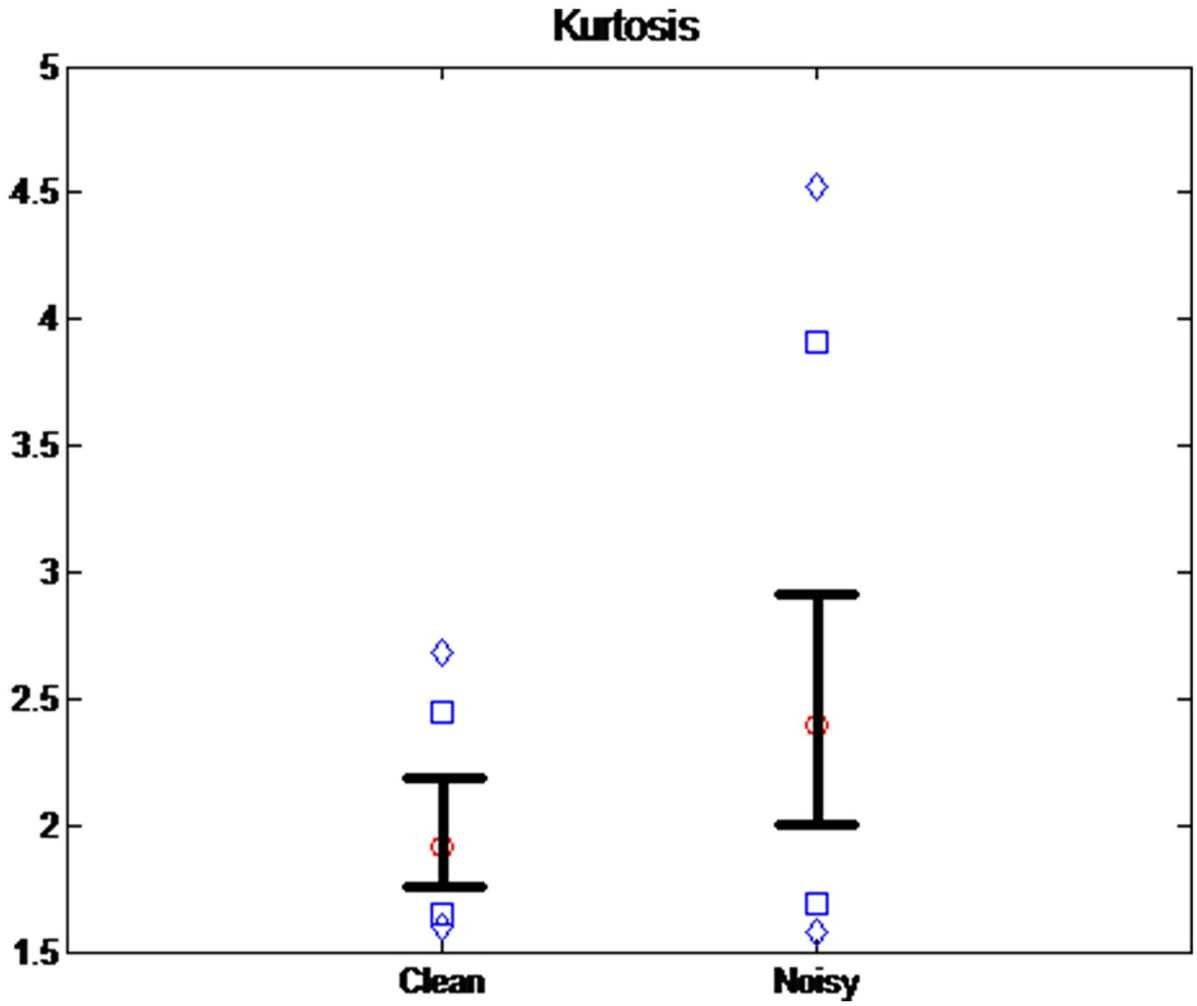
The distribution of Kur from the clean and noisy signal groups. The diamonds above and below represent the 5^th^ and 95^th^ percentiles of each group, and the squares above and below represent the 90^th^ and 10^th^ percentiles. Whiskers above and below represent the 75^th^ and 25^th^ percentiles, respectively. The circle is the median value.

Where *μ*_*4*_ is the fourth moment of the mean and *σ* is the standard deviation. Kur represents a heavy tail and peakedness, or a light tail and flatness, of a distribution relative to the normal distribution. Fig 2 shows the Kur value distribution from clean and noisy signal groups. The diamonds above and below represent the 5^th^ and the 95^th^ percentiles of each group, and the squares above and below represent the 90^th^ and the 10th percentiles. Whiskers above and below represent the 75^th^ and the 25^th^ percentiles, respectively. The circle is the median value. The mean ± standard deviation was 1.9973 ± 0.3257 for a clean signal and 2.6206 ± 0.9446 for a noisy signal. This shows that the Kur mean value of a noisy signal is 1.31 times higher than that of a clean signal. In addition, there was a significant difference between the two groups at p < 0.01 based on ANOVA and Bonferroni’s t-test. Then, the condition for motion artifact detection is based on each threshold value of TH_tpr_ and TH_se_: TPR ≥ TH_tpr_ and Kur ≥ TH_kur_.

### Post-Exercise in the Cardiac Rehabilitation Program

Upon completion of the CR exercise, the patient completes a questionnaire using scales for chest pain, dyspnea, and leg pain during the exercise. The information from the questionnaire is stored in the app along with the achieved ratio of measured HR to THZ, which can be used as objective and subjective indicators for evaluating exercise. The post-exercise information is recorded with pre-exercise set-up information, such as THZ, exercise type, exercise stage order, and duration of each stage, to indicate the exercise history, which can be monitored by the patient and potentially also by clinicians.

### Data Availability

The data used for this work have been uploaded on Figshare website. (https://figshare.com/articles/CRP_all_raw_data_final_xlsx/3438878/2)

## Result

### Experimental Results on Threshold Values *TH*_*tpr*_ and *TH*_*kur*_ and HR Estimation

**Fig 3:**
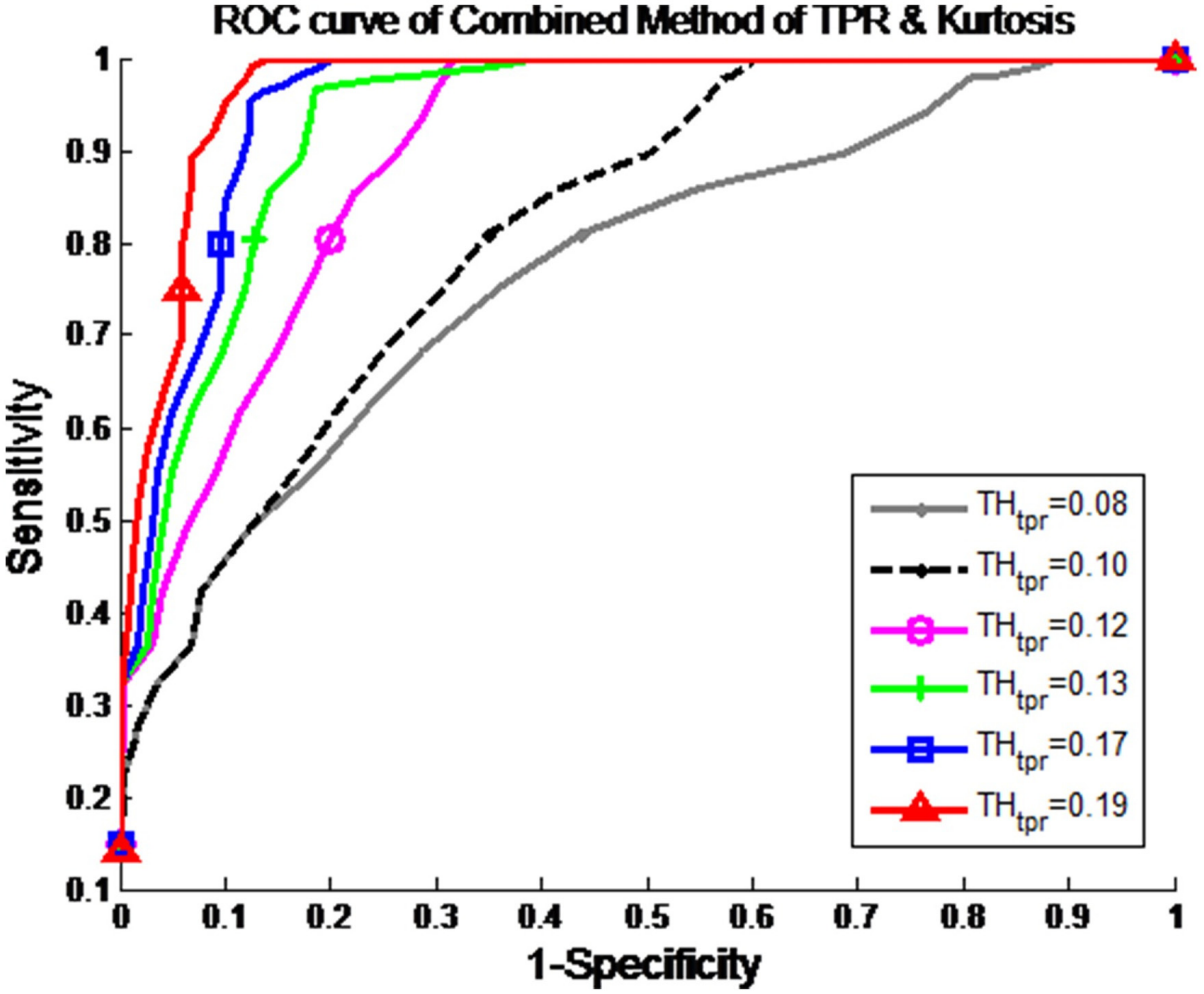
The ROC curves. Receiver operating characteristic (ROC) curves with 1—specificity versus sensitivity for TH_tpr_ and TH_kur_, where a noisy signal corrupted by motion artifact is identified when TPR ≥ TH_tpr_ and Kur ≥ TH_kur_.

Fig 3 shows the receiver operating characteristic (ROC) curves with 1 − specificity versus sensitivity for TH_tpr_ and TH_kur_, where a noisy signal corrupted by motion artifacts is identified when TPR ≥ TH_kur_ and Kur ≥ TH_kur_. For the ROC evaluation, we determined the numbers of true positive (TP), true negative (TN), false positive (FP), and false negative (FN) results. Subsequently, we calculated the sensitivity TP/(TP+FN), specificity TN/(TN+FP), and accuracy (TP+TN)/(TP+TN+FP+FN). We found that the optimum threshold values were TH_tpr_ = 0.19 and TH_kur_ = 1.7, which provide sensitivity of 0.8227, specificity of 0.9364, and accuracy of 0.8795.

Subsequently, we filtered out the detected noisy segments based on the condition TPR ≥ TH_tpr_ and Kur ≥ TH_kur_, and evaluated the HR estimation for the rest of the segments, which were defined as the clean signal segments. Note that if a segment is found to include a noisy signal, its HR value is not provided to the patient.

To evaluate the estimated HR values, we used mean absolute error (MAE) and root mean squared error (RMSE), defined as follows:

$$\text{Mean Absoulte Error }(\text{MAE})=\;\sum^{\text{​}}\frac{\left|Y_{iPhone}-Y_{holter}\right|}{N}$$ (5)

$$\text{Root Mean Squared Error }\left(\text{RMSE}\right)=\;\sqrt{\sum^{\text{​}}\frac{{\left(Y_{iphone}-Y_{holter}\right)}^{2}}{N}}$$ (6)

where *Y*_*iphone*_ is the HR (bpm) estimated from the iPhone at the *i*^*th*^ segment, and *Y*_*holter*_ is the HR (bpm) from the Holter at the *i*^*th*^ segment. Table 3 summarizes the RMSE and MAE values for walking (warm-up), two stages of jogging, and walking (cool-down).

**Table 3:** Root mean square error (RMSE) and mean absolute error (MAE) for walking (warm-up), two stages of jogging, and walking (cool-down).

Error	Walking (warm-up)	Jogging	Additional jogging	Walking (Cool-down)
**RMSE (bpm)**	2.92	7.16	7.67	4.37
**MAE (bpm)**	2.41	4.81	5.23	3.70

In the walking for warm-up, RMSE and MAE were 2.92bpm and 2.41bpm, respectively. In the walking for cool-down, RMSE and MAE were 4.37bpm and 3.70bpm, respectively. In the first jogging session, RMSE and MAE were increased to 7.16bpm and 4.81bpm, respectively. In the additional jogging session, which exercise intensity was slightly increased, RMSE and MAE were also slightly increased, to 7.67bpm and 5.23bpm. These high rates mainly stem from the noisy signal segments corrupted by motion artifacts. Most noisy segments were filtered out, but a few remained. As shown in Table 4, the highest rate of FNs was found in the additional jogging session, followed by jogging session, cool-down, and warm-up.

**Table 4:** Number of segments, noisy segments, and false negatives in walking (warm-up), two stages of jogging, and walking (cool-down).

Segments	Walking (warm-up)	Jogging	Additional jogging	Walking (Cool-down)
**Total segments #**	4,800	9,600	9,600	4,800
**Noisy segments #**	951	2,872	3,753	1,154
**False negatives #**	92	514	788	154

### Cardiac Rehabilitation Application

**Fig 4:**
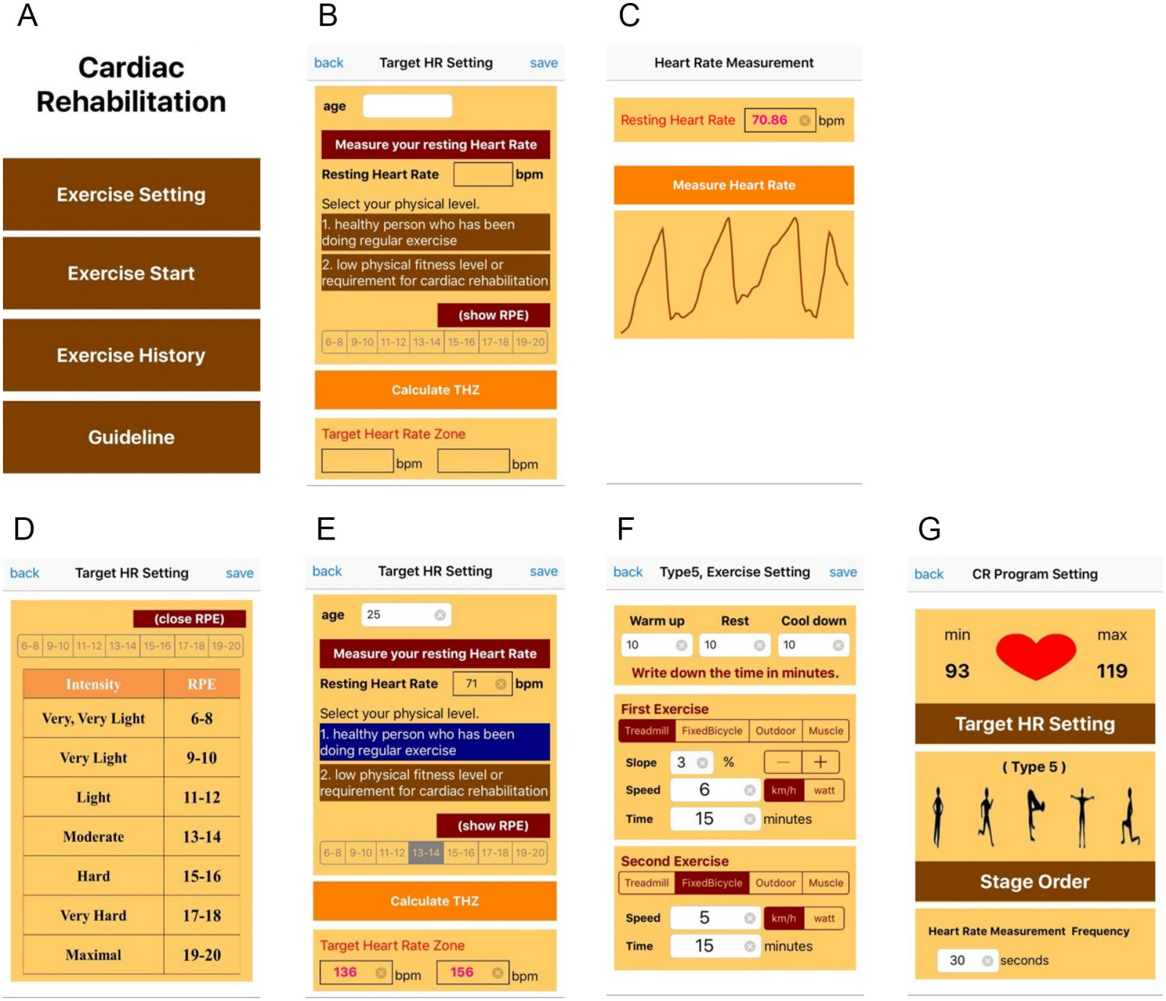
Pre-exercise on the developed cardiac rehabilitation program (CRP) with an iPhone app. (a) main menu, (b) target heart rate zone (THZ) set-up, (c) exercise type, stage order and heart rate (HR) estimation, and (d) duration of each stage and specification.

Fig 4(a) shows the CRP menu, which includes an “Exercise Setting” button for pre-exercise, an “Exercise Start” button for CR exercise, an “Exercise History” button for post-exercise, and a “Guideline” button for CR exercise introduction and instruction. Fig 4(b)–4(g) shows the pre-exercise for THZ, exercise type, exercise stage order, duration of each stage, and HR estimation frequency, which can be set up by the patient. For the THZ calculation, as shown in Fig 4(b), the patient inputs their age and enables measurement of their pulse by placing their fingertip on the smartphone camera for the calculation of resting HR. As shown in Fig 4(c), the patient allows their pulse to be measured for 2s, from which the resting HR is estimated. Subsequently, the patient selects their physical level: 1) a healthy person who has regularly been performing exercise, or 2) a person with a low physical fitness level or requiring cardiac rehabilitation. Finally, the patient selects RPE, representing exercise intensity. To promote understanding of this variable, the patient can view the RPE scale and its exercise intensity, as shown in Fig 4(d). Based on age, resting HR, and RPE, the THZ is automatically calculated for the CR exercise, as shown in Fig 4(e). Alternatively, the THZ can be determined based on the ETT performed by clinicians and manually entered. Upon completion of the THZ set-up, the exercise stage order, HR estimation frequency, duration of each stage, exercise type, and its specifications, such as slope and speed, can be set up as shown in Fig 4(f) and 4(g).

**Fig 5:**
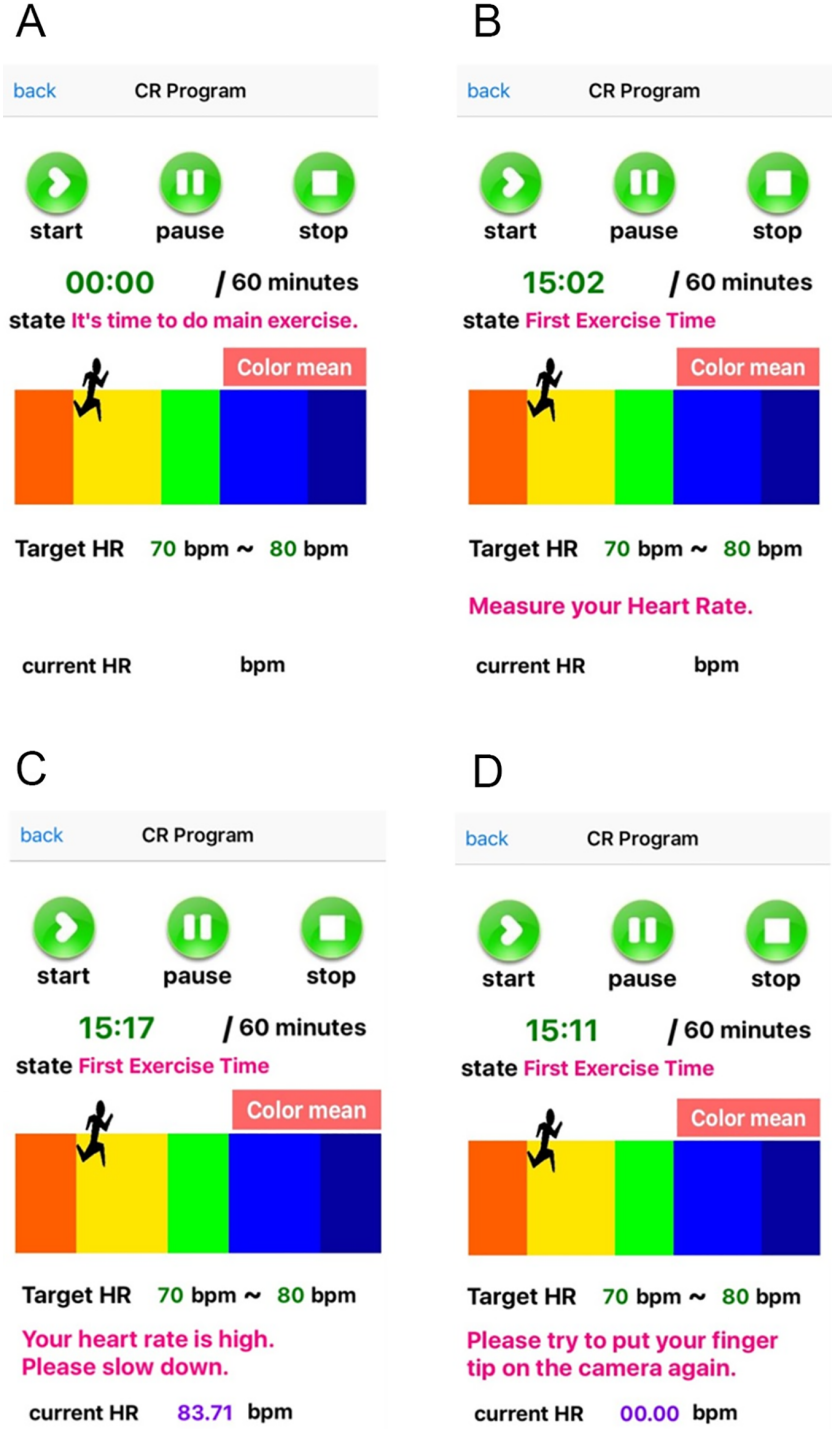
Exercise with the S-CRP on an iPhone. (a) shifting to the main exercise stage with voice guidance: “It is time to do the main exercise,” (b) measuring HR with voice guidance: “Please place your fingertip on the camera to estimate your heart rate,” (c) providing guidance on the exercise intensity with voice guidance: “Your heart rate is high. Please slow down,” and (d) detecting motion artifacts: “Your measured pulse is of low quality. Please place your fingertip on the camera again.”

Fig 5 shows examples of the main exercise pages on the S-CRP. In Fig 5(a) the S-CRP informs the patient of the exercise stage transition with the voice guidance: “It is time to do the main exercise” when the exercise stage shifts from rest to the main exercise stage. In Fig 5(b), the S-CRP informs the patient of HR measurement with the voice guidance: “Please put your fingertip on the camera to estimate your heart rate.” In Fig 5(c), the S-CRP informs the patient of the exercise intensity with the voice guidance: “Your heart rate is high. Please slow down.” In Fig 5(d), the S-CRP informs the patient of the need to measure the HR again due to motion artifacts with the voice guidance: “Your measured pulse is of low quality. Please place your fingertip on the camera again.” In this way, during exercise, voice guidance on exercise status, HR estimation, and exercise intensity is provided to the patient.

**Fig 6:**
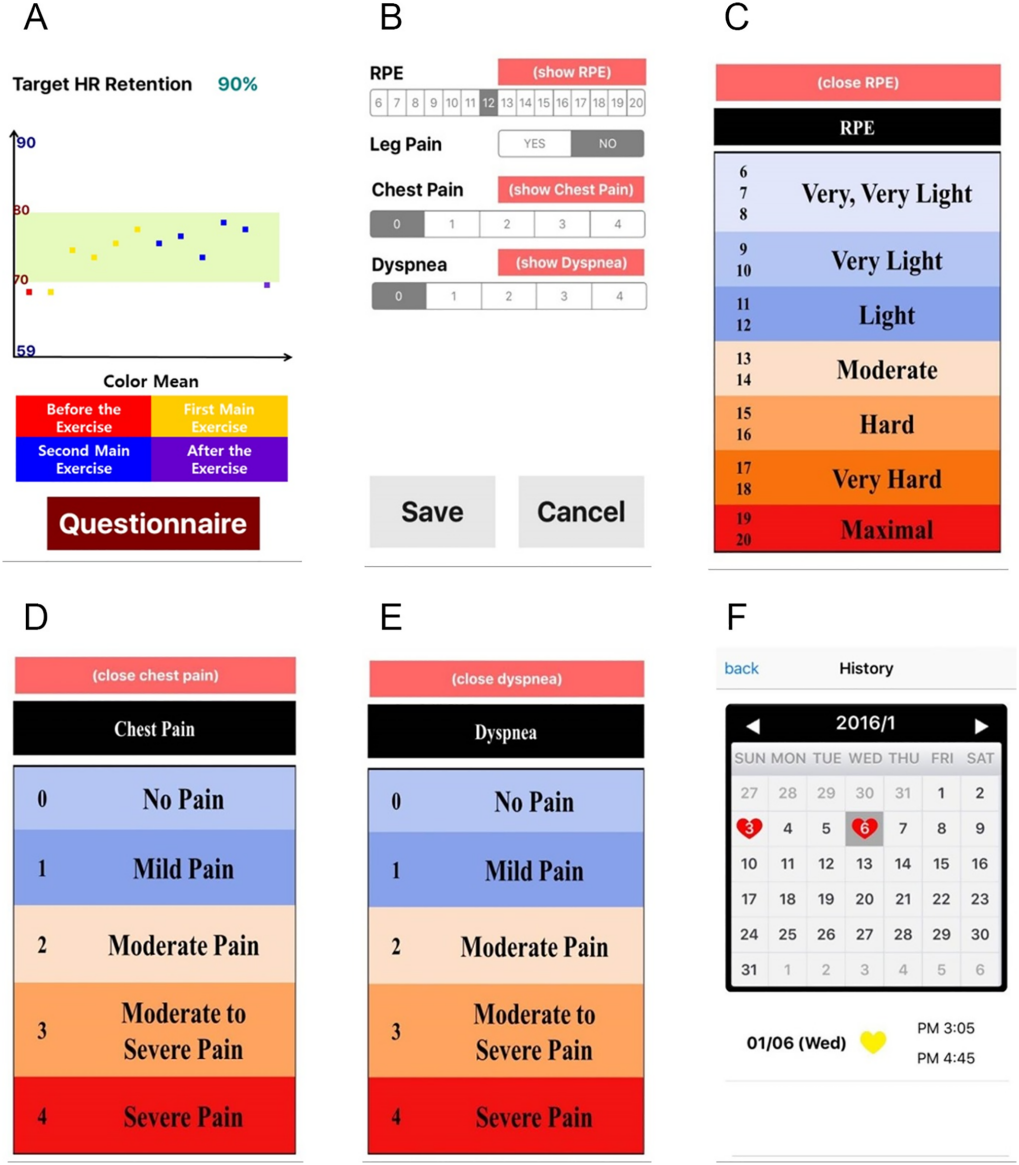
Rehabilitation results and medical report.

Fig 6 shows the post-exercise pages on the S-CRP. In Fig 6(a), the S-CRP provides information on the achieved ratio of the measured HR to the THZ by plotting these two variables. The patient can monitor how efficiently they performed the exercise as an objective evaluation. In Fig 6(b), the questionnaire on the patient’s post-RPE (recognition of exercise intensity), chest pain, leg pain, and dyspnea is shown. The patient completes the questionnaire as a form of subjective evaluation. To promote their understanding, the patient can view the descriptions of post-RPE, chest pain, and dyspnea, as shown in Fig 6(c)–6(e), respectively. Finally, the patient can monitor their previous exercise information using a calendar. A heart marks each day when a patient exercised. If a patient clicks a date marked with a heart, they can see all of the available information on the performed exercise. Clinicians can also monitor the patient’s previous exercise patterns.

## Discussion

We introduced an S-CRP that indicates an appropriate level of exercise for keeping the HR within the THZ by utilizing only a smartphone, without the need for any external devices. Our proposed S-CRP can improve patient health by ensuring a more active life, and promote a return to a normal life in a timely manner. For future research, we are recruiting more subjects, including patients with cardiac disease and healthy subjects, to validate our developed S-CRP, which is under IRB preparation. The S-CRP can be modified to also include lifestyle changes, education, and emotional support for more efficient cardiac rehabilitation.

The developed S-CRP is available when a patient is still in hospital after undergoing treatment for a heart attack or other related cardiac disease. It is also available once a patient has left hospital or at any other time to help prevent future heart problems. Furthermore, it is available for healthy individuals, for whom it can ensure efficient exercise by indicating the appropriate exercise intensity.

**Fig 7:**
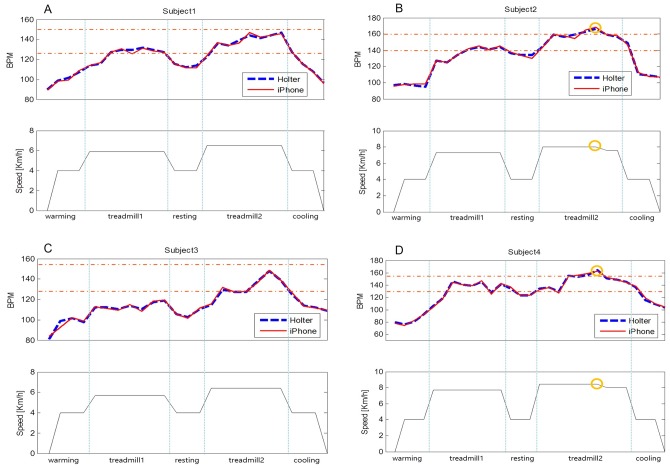
Individual HR traces obtained from the Holter and the S-CRP during exercise: the voice guidance “Your heart rate is high. Please slow down” was given to the participant #2 and #4 (a circle mark). (a) participant #1, (b) participant #2, (c) participant #3, and (d) participant #4.

To further validate our developed S-CPR, we applied to a maximal exercise testing with the resultant individual *HR*_*max*_. As a pilot study, four healthy participants first performed on the maximal exercise testing, which considers metabolism (METs), HR, blood pressure, respiratory exchange ratio (RER) and RPE, and prescribes the exercise intensity including *HR*_*max*_ using Q-Tel RMS program (Mortara Inc., Milwaukee, WI, USA) [2,19,20]. Written informed consent was obtained from the participants for publication of this case report and any accompanying analysis. Subsequently, each participant performed the prescribed exercise which consists of 2 min of walking for a warm-up, 5 min of jogging, 2 min of rest, an additional 5 min of jogging, and 2 min of walking for cool-down, all on a treadmill. In the S-CRP, the THZ was set within 70% of the individual HRmax (THZ < 0.7 *HR*_*max*_), and the frequency of HR estimation was set to 30 sec. Table 5 shows the individual exercise prescription on treadmill’s speed, slope and time provided by a cardiac rehabilitation specialist. To evaluate the HR estimation accuracy from a smartphone, ECG was simultaneously recorded using a 24-hour Holter monitor (SEER Light, GE Healthcare, Milwaukee, WI, USA). Fig 7(a) to 7(d) show the HR traces obtained from the Holter and the S-CRP (iphone 6) for each participant during the exercise. The participants #1 and #3 performed the exercise by keeping the individual THZ without the aid of S-CPR voice guidance as shown in Fig 7(a) and 7(c). On the other hand, for the participants #2 and #4, the voice guidance provided the following message: “Your heart rate is high. Please slow down” as shown in Fig 7(b) and 7(d). Then, the treadmill speed was slowed down by 0.5 km/h to keep the HR within THZ. In the walking for warm-up, RMSE and MAE were 2.36bpm and 1.93bpm, respectively. In the walking for cool-down, RMSE and MAE were 1.40bpm and 1.00bpm, respectively. In the first jogging session, RMSE and MAE were 1.21bpm and 0.95bpm, respectively. In the additional jogging session, RMSE and MAE were 1.83bpm and 1.43bpm.

**Table 5:** Exercise prescription based on a maximal exercise testing.

Exercise Prescription	Maximum Range			
	Heart Rate (BPM)	Speed (km/h)	Slope (%)	Main exercise time (min)
**Participant #1**	151	6.5	4	5
**Participant #2**	161	8	5	5
**Participant #3**	159	6.4	3	5
**Participant #4**	153	8.4	5	5

One key issue is to accurately estimate HR. To increase the HR estimation accuracy, we may increase the measurement time. However, this would not only result in inaccurate results when HR fluctuates, but also cause the patient discomfort. To minimize such discomfort, we instead periodically measured the pulse signals from a fingertip on a smartphone camera for a short time (2 s). Since a short signal is more sensitive to motion artifacts, we established a system for automatically detecting motion artifacts. As introduced in this paper, we measured the pulse signals again if the signal was found to be corrupted by motion artifacts, which were automatically detected. To increase the accuracy of motion artifact detection, more statistical methods can be added to TPR and kurtosis, as we proposed. We performed testing with additional statistical methods such as sample entropy, variance, skewness, and root mean square successive difference, and found that the accuracy only increased by less than 2%. To dramatically increase the accuracy, more research should focus on detecting motion artifacts during exercise to a clinically acceptable level.

The introduced S-CRP enables appropriate exercise with a short HR measurement time and automatic voice guidance, but some patients may still feel uncomfortable because they have to hold a smartphone while exercising. Recently, many wearable devices, from Apple, Fitbit, Samsung, and other companies with the capacity to measure HR in real time have been released. The proposed S-CRP can be incorporated into these existing wearable devices, which may provide a more convenient CRP without the need to hold a smartphone. We are also developing and testing a wearable device for dedicated CRP, which we hope to report on in the near future.

## Conclusion

We introduced an S-CRP that utilizes only a smartphone, without the need for any external devices. For efficient cardiac rehabilitation exercise, we developed an application to automatically guide the intensity of exercise by comparing the estimated HR with the THZ. The HR was estimated by using video images of a fingertip taken by the smartphone’s built-in camera. For highly accurate HR estimation, we combined TPR and kurtosis, and filtered out the signals with motion artifacts. We believe that our proposed S-CRP could gain wide acceptance as a home-based clinical CRP tool with the advantages of good accessibility, low cost, and ease of use.
